# Interface-Induced Synaptic Performance in CeO_2_/La_0.8_Ba_0.2_MnO_3_ Oxygen Reservoir Junction

**DOI:** 10.1021/acsami.5c19731

**Published:** 2025-12-10

**Authors:** K. N. Rathod, Gopal Datt, Bagher Aslibeiki, Ted Johansson, Gianni Barucca, Davide Peddis, M. Venkata Kamalakar, Tapati Sarkar

**Affiliations:** † Division of Solid-State Physics, Department of Materials Science and Engineering, 8097 Uppsala University, Uppsala, SE 751 03, Sweden; ‡ Division of X-ray Photon Science, Department of Physics and Astronomy, Uppsala University, Uppsala, SE 751 20, Sweden; § Faculty of Physics, 56947 University of Tabriz, Tabriz 51666-16471, East Azerbaijan Province, Iran; ∥ Division of Solid-State Electronics, Department of Electrical Engineering, Uppsala University, Uppsala, SE 751 03, Sweden; ⊥ Department of Science and Engineering of Matter, Environment and Urban Planning, University Politecnica delle Marche, Via Brecce Bianche 12, 60131 Ancona, Italy; # Department of Chemistry and Industrial Chemistry & Genova INSTM RU, nM2-Lab, University of Genova, 16146 Genova, Italy; ∇ National Research Council, Institute of Structure of Matter, nM2-Lab, Via Salaria km 29.300, Monterotondo, Scalo 00015, Roma, Italy

**Keywords:** manganite, oxygen vacancy, interface engineering, memristor, synapse, potentiation, depression, neuromorphic

## Abstract

Realizing next-generation intelligent applications requires novel resistive switching devices that can operate with low power, high stability, and desired neuromorphic performance. La_0.8_Ba_0.2_MnO_3_ (LBMO), a functional complex oxide exhibiting a room-temperature metal–insulator transition, shows promise in this context. In this work, we demonstrate interface-engineered resistive switching in the LBMO thin film junction by introducing an ultrathin CeO_2_ insertion layer. Compared to bare LBMO film, which requires higher forming voltages and suffers from limited stability and large cycle-to-cycle variability, the CeO_2_/LBMO (LBC) device exhibits stable, low-power bipolar resistive switching. The LBC device achieves a low forming voltage of 2.2 V, an ON/OFF ratio of ∼10^2^, endurance of 600 switching cycles, and data retention of 10^3^ seconds. The improved performance is attributed to controlled oxygen vacancy migration and redistribution facilitated by the CeO_2_ interlayer. Furthermore, the LBC device displays, for the first time, bioinspired synaptic behaviors, such as gradual potentiation and depression under pulsed stimuli, and exhibits linear plasticity under nonidentical pulse schemes, effectively emulating synaptic weight modulation. Our results demonstrate an interface-induced resistive switching device as a compelling candidate for next-generation neuromorphic components.

## Graphical abstract

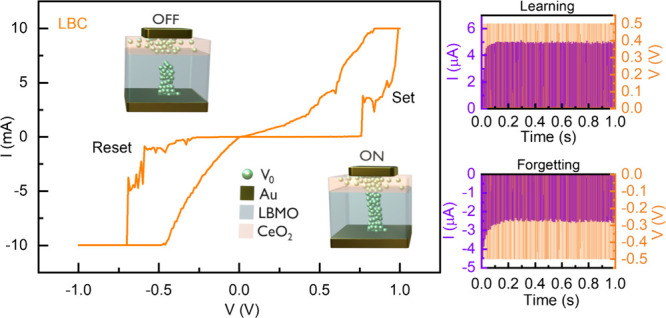

## 1 Introduction

Inventing novel neuromorphic computing components that emulate biological synapses and neurons, and their scalable fabrication, can dramatically alter the conventional von Neumann architecture-based computing landscape. In this context, resistive random-access memory (RRAM), based on a resistive switching mechanism with nonvolatility, scalability, fast switching, and low power consumption, shows synapse-emulating ability.^1^ As resistive switching technologies continue to grow and mature, scalable and environmentally sustainable application-specific RRAM systems become a possibility.^2–6^ Park et al.^2^ investigated a filament-free trilayer RRAM (AlO_3_/TiO_2_/TiO_*x*_) that would operate as low-energy neuromorphic computing, specifically for effective edge applications. A bioinspired SiO_*x*_ RRAM allows for experience-based learning with improved robustness for autonomous navigation.^3^ Memory devices using Ipomoea carnea have been developed to provide a means for sustainable and biocompatible alternatives.^6^ Meanwhile, state-of-the-art advancements indicate the promise of RRAM for high-density integration, an architectural alignment with neural networks, and potential for more significant energy efficiencies over traditional complementary metal-oxide-semiconductor (CMOS)-based systems.^7–9^ Despite this huge promise, the central challenge here remains, i.e., controlling the variability of the performance parameter to achieve stable and uniform resistive switching across large-size devices. Primarily, methods such as material modification through doping and control of oxygen concentration,^10^ innovations in structural design^11^ have been employed to maximize resistive switching performance. Here, functional complex oxides such as manganites have been promising,^12^ particularly La_1–*x*_Ba_*x*_MnO_3_, since it exhibits resistive switching. The concentration of doping in La_1–*x*_Ba_*x*_MnO_3_, especially when x = 0.2, is significant because it promotes a metal-to-insulator transition close to room temperature.^13^ However, the resistive switching mechanism in La_0.8_Ba_0.2_MnO_3_ (LBMO) remains ambiguous and limited.^14–17^ Insertion of an oxygen exchange layer, which can act as a reservoir in a memristor device structure, is a promising means to overcome the unstable resistive switching performance issue.^18^ Recent advances have shown effective strategies for oxygen-reservoir and interface engineering that improve resistive switching. For example, HfO_2_:CeO_2_ nanocomposites enable operation without the need for electroforming,^19^ CeO_2_/Nb-SrTiO_3_ heterojunctions demonstrate gradual analog modulation,^20^ and Ta_2_O_5_-based bilayers achieve low-power multilevel switching.^21^ These studies highlight the increasing importance of controlling oxygen at interfaces in oxide-based memristors. The insertion layer could lead to ideal synaptic behavior and scalable memristive circuits. However, this has not been done before in LBMO-based devices.

In this work, we designed a new CeO_2_/LBMO (LBC) device that introduces a CeO_2_ insertion layer as an oxygen reservoir for efficient switching, inducing reliable synaptic behavior in LBMO. In addition to detailed transport measurements that show enhanced memristive behavior and time-resolved measurements that show learning and unlearning, electronic structure characterization helps us to determine oxygen stoichiometry and relate it to the device performance, unveiling new insights into understanding the conduction mechanisms, where Ce^3+^ and Ce^4+^ redox processes modulate oxygen vacancies in the CeO_2_ insertion layer.

## 2 Experimental Section

### 2.1 Device Fabrication

We have used CMOS-compatible n-type Si substrates (5 × 5 mm^2^) with a ∼ 300 nm SiO_2_ layer to deposit thin films of LBMO. The SiO_2_/Si substrates were ultrasonically cleaned in acetone and isopropyl alcohol for 10 min each to remove organic impurities. A 10 nm Ti adhesion layer and 90 nm Au bottom electrode were deposited by electron beam evaporation (1.5 Å/sec). The LBMO films were deposited via pulsed laser deposition (PLD) using a Lambda-Physik Compex 205 KrF excimer laser (248 nm, 1.8 J/cm^2^, 10 Hz) with a substrate temperature of 300 °C, substrate-target distance at 50 mm, and an oxygen partial pressure of 1 × 10^–3^ mbar. A 15 nm thick CeO_2_ layer was deposited on one of the films using PLD (2.3 J/cm^2^, 2 Hz) with an oxygen pressure of 0.4 mbar and a substrate temperature of 300 °C. Thinner films of CeO_2_ may lead to discontinuity and random switching. In contrast, thicker films increase forming and set voltages while reducing efficiency. We aimed for a 15 nm CeO_2_ layer for continuous coverage over LBMO grains, which aligns with previous literature.^22^ This choice ensures a uniform oxygen-vacancy reservoir without pinholes or high series resistance. A shadow mask was used to pattern the gold bottom electrode step, and top Au electrodes were deposited similarly.

### 2.2 Device Characterizations

Grazing incidence X-ray diffraction (GIXRD) was obtained using a Siemens D5000 X-ray diffractometer in parallel beam geometry at a grazing angle of 1°, and peaks matched with JCPDS 04–016–8655 (LBMO) and 04–005–6041 (CeO_2_). Transmission electron microscopy (TEM) and scanning electron microscopy (SEM) were performed using a Philips CM200 microscope operating at 200 kV and equipped with a LaB6 filament, and a Tescan Vega 3 microscope, respectively. For cross-sectional TEM observations, samples were prepared using a conventional procedure involving the fabrication of a ″sandwich″ structure, followed by slicing, mechanical polishing with sandpaper and diamond pastes, and subsequent thinning with a dimple-grinder. Final thinning was carried out using an ion beam system (Gatan PIPS) with Ar^+^ ions at 5 kV. Atomic force microscopy (AFM) was conducted using a Bruker Dimension Icon ICON4-SYS in the tapping mode over a 1 × 1 μm^2^ area to assess surface roughness and uniformity. The X-ray photoelectron spectroscopy (XPS) analysis was carried out on a Physical Electronics PHI Quantera II instrument using a monochromatic Al Kα (1486.6 eV).^23^ Survey scans were performed in the binding energy range of 0–1000 eV. High-resolution element-specific XPS scans were accomplished for manganese, oxygen, and cerium. Electrical measurements were carried out using an Agilent B1500A semiconductor device parameter analyzer, and an Agilent B1531A unit was used for pulse measurements.

## 3 Results and Discussion

### 3.1 Morphology and Chemical Composition

Figure 1a shows the schematics of LB (LBMO without CeO_2_, top) and LBC (bottom) thin film devices. As shown in Figure 1b, GIXRD shows a textured polycrystalline structure of both films without detectable secondary phases. The AFM images show that both LB and LBC films (Figure 1c,d) have relatively smooth and flat surfaces, with the LBC film (Figure 1d) exhibiting slightly higher roughness ∼ 8 nm (∼5 nm for LB film), suggesting the effect of the CeO_2_ layer on the film morphology. Overall, these films provide a robust platform to fabricate devices in the scheme of Figure 1a. A cross-sectional TEM image (Figure 2a) of the LBC device reveals the different layer thicknesses in the stacked structure consisting of CeO_2_ layer (∼15 nm), LBMO (∼140 nm), Au/Ti electrodes (∼90 nm), and the SiO_2_ layer (∼300 nm), in good agreement with the expected layer thicknesses. A top-view SEM image of the LB device is shown in Figure 2b, showing also the electrodes.

**Figure 1:**
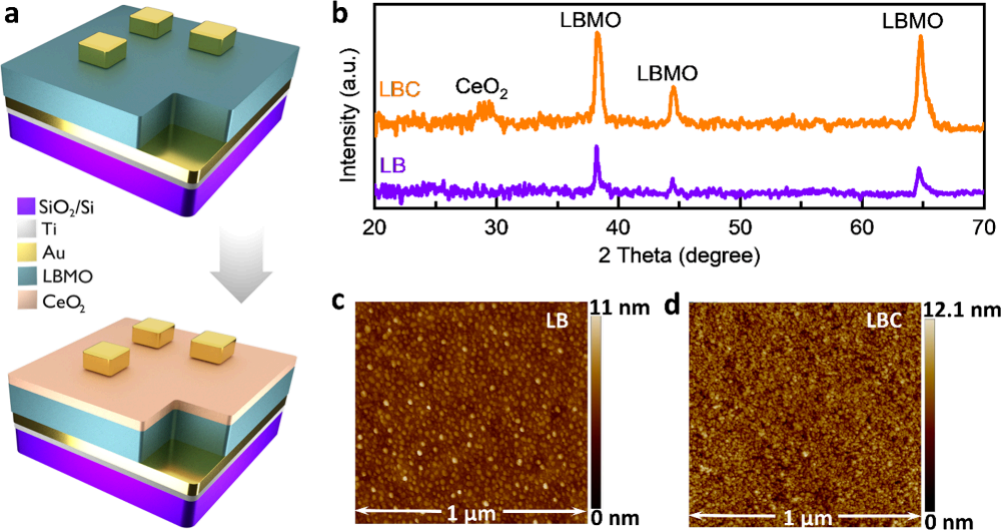
Device schematic of (a) LB (top) and LBC (bottom) structures; (b) GIXRD patterns of the LB and LBC devices; AFM images of (c) LB surface and (d) LBC surface.

**Figure 2:**
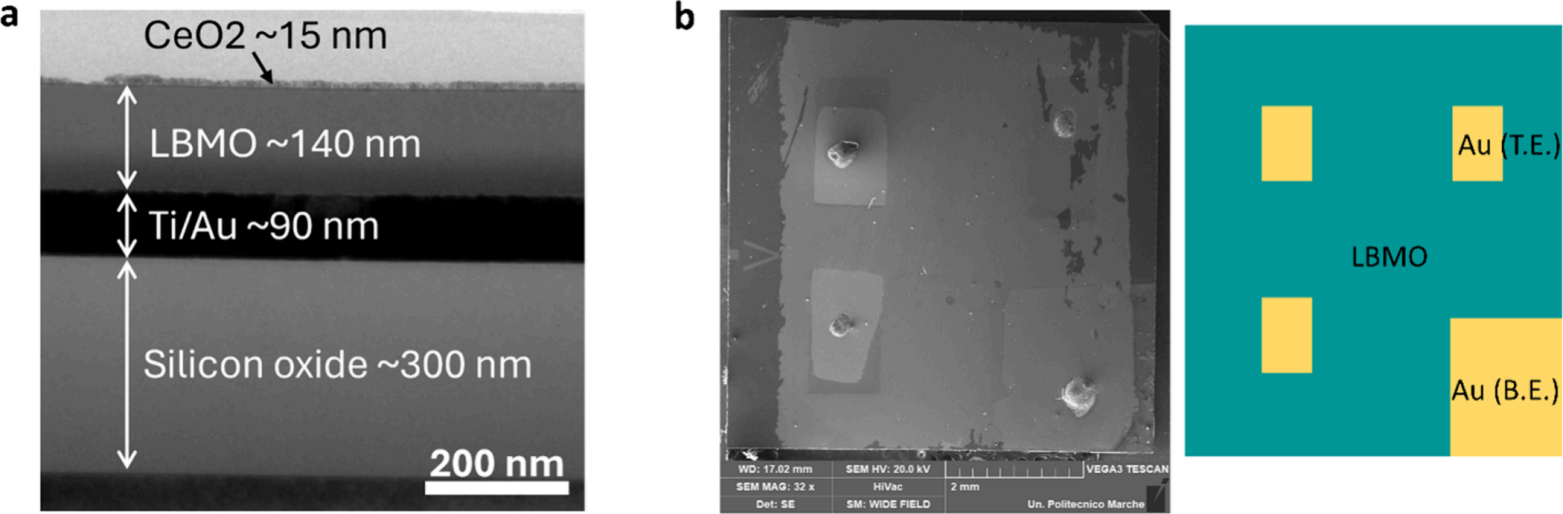
(a) Cross-sectional TEM image of LBC device showing the layers in the stacked structure and (b) top-view SEM image of LB device with the sketch of the Au top (T.E.) and bottom (B.E.) electrodes.

To assess the electronic states and defect chemistry, XPS analysis of Mn, O, and Ce ions in the LB and LBC films was carried out (Figure 3). Ar^+^ ion presputtering (500 V, 30 s) confirmed the removal of surface contamination, i.e., adventitious carbon, without disrupting the films’ underlying electronic and chemical states. This treatment was uniformly applied to the LB and LBC samples to ensure a direct comparison of surface and near-surface chemistries. As shown in Figure 3a, the survey scans display the main peaks of each element, denoted with dashed lines. The element-specific spectra were fitted using Tougaard background and LA (1.53, 243) Voigt line shapes with baseline correction, as shown in Figure 3b–f. Figure 3b shows the deconvoluted Ce 3d high-resolution spectrum for the LBC film, indicating the spin–orbit splitting of Ce 3d core-level into Ce 3d_5/2_ and Ce 3d_3/2_ components. Peak assignments for Ce^3+^ and Ce^4+^ ions are consistent with prior studies,^24,25^ validating the analysis and allowing quantitative estimation of the cerium oxidation state distribution. The XPS fitting of Ce 3d with R^2^ = 98.26% (Table 1) shows the complexity of ceria’s multipeak spectrum, with reliable oxidation state quantification. Localized states within the band gap are achieved by a partially filled 4f^1^ configuration in Ce^3+^. 4f^0^ configuration of Ce^4+^ contributes indirectly to the band structure by hybridization with Ce 5d and O 2p orbitals. The Ce^3+^ ion concentration, found to be 43.4% (Table 2), is more than the typical stoichiometry of CeO_2_, indicating deviation from ideal lattice stoichiometry. This affects the defect chemistry because such a 4f^1^ configuration can play the role of electronic reservoirs, creating a space for charge compensation and transport processes. This high Ce^3+^ content directly correlates with oxygen vacancy formation, governed by the following reaction:

1 $$2Ce^{4+}+O_{L}^{2-}\to 2Ce^{3+}+\frac{1}{2}O_{2}\;\left(g\right)+V_{O}^{..}$$

**Figure 3:**
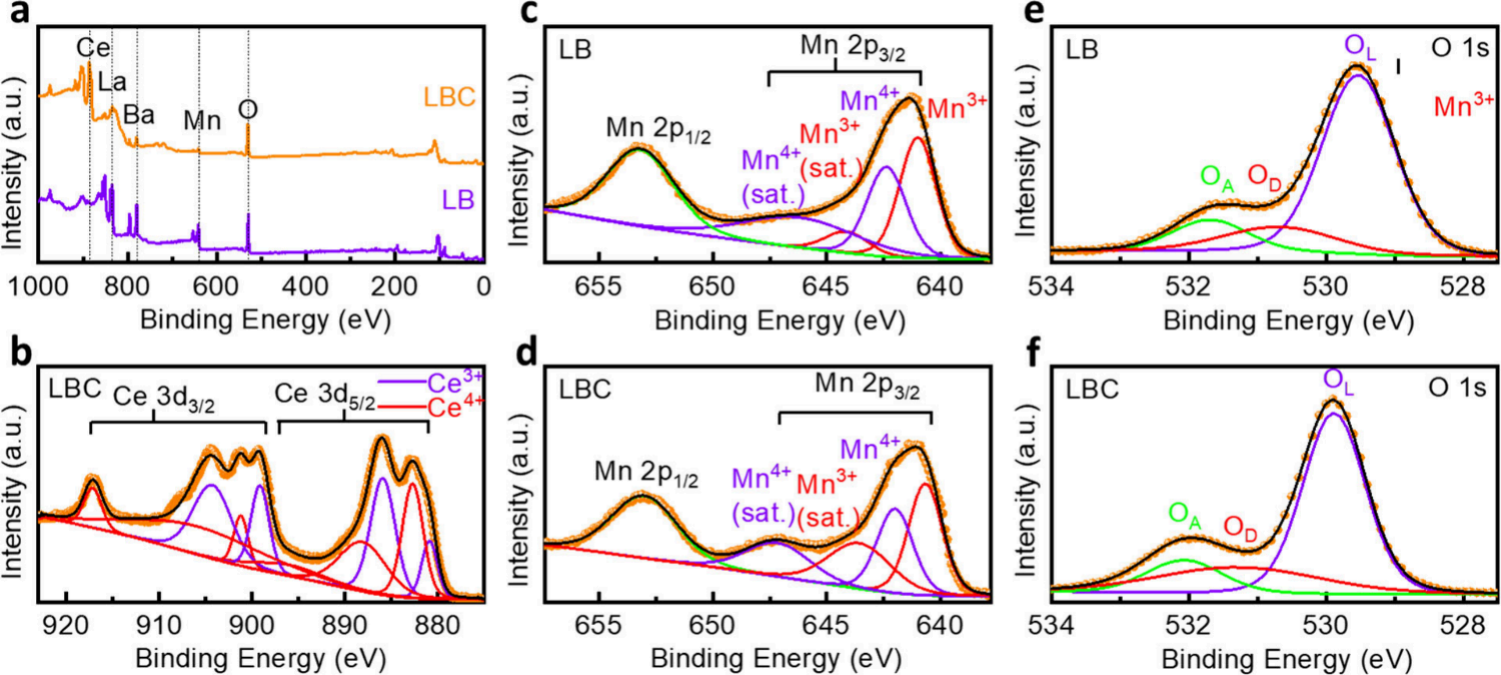
(a) XPS survey scans of LB and LBC devices, (b) Ce 3d core-level spectrum of LBC, Mn 2p core-level spectra of (c) LB and (d) LBC, O 1s core-level spectra of (e) LB and (f) LBC. Satellite peaks are labeled as “sat.”, with O_L_ representing lattice oxygen, O_D_ indicating oxygen vacancy defect, and O_A_ denoting adsorbed O-species.

**Table 1:** XPS Core-Level O 1s, Mn 2p, and Ce 3d Peak Positions

Sample	Element	Feature	Position	R^2^
LB	O 1s	O_L_	529.53	99.97%
		O_W_	531.69	
		O_D_	530.69	
	Mn 2p_3/2_	Mn^3+^	640.99	99.72%
		Mn^3+^ sat.	644.12	
		Mn^4+^	642.37	
		Mn^4+^ sat.	646.20	
	Mn 2p_1/2_	-	653.19	
LBC	O 1s	O_L_	529.88	99.98%
		O_W_	532.07	
		O_D_	531.28	
	Ce^3+^ 3d_5/2_	v_0_	880.92	98.26%
		v′	885.93	
	Ce^4+^ 3d_5/2_	v	882.72	
		v″	888.12	
		v‴	896.00	
	Ce^3+^ 3d_3/2_	u_0_	899.15	
		u′	904.30	
	Ce^4+^ 3d_3/2_	u	901.22	
		u″	905.88	
		u‴	917.15	
	Mn 2p_3/2_	Mn^3+^	640.66	99.62%
		Mn^3+^ sat.	643.57	
		Mn^4+^	641.99	
		Mn^4+^ sat.	647.15	
	Mn 2p_1/2_	-	653.01	

**Table 2:** Elemental Concentrations along with Integrated Intensity Ratios Obtained from XPS Fitting

Sample	Mn^4+^/Mn^3+^	O_L_/O_D_	Ce^3+^ (%)	Ce^4+^ (%)
LB	0.80 ± 0.13	4.60 ± 0.55	-	-
LBC	0.84 ± 0.19	2.65 ± 0.21	43.40	56.60

Here, O_L_^2–^ represents lattice oxygen and V_O_^..^ denotes a doubly ionized oxygen vacancy. Two electrons that support the conversion of two Ce^4+^ ions into two Ce^3+^ ions are donated by the emission of lattice oxygen as molecular oxygen $\frac{1}{2}O_{2}\;\left(g\right)$ . This transformation highlights the oxygen vacancies as (1) charge compensators to stabilize the lattice and (2) contributors to increase the electronic conductivity by defect-state formation in the band gap. In the presence of Ce^3+^, oxygen vacancy formation is favored, which leads to a local distortion of the lattice. Previously empty Ce 4f orbitals then get occupied by electrons introduced by oxygen vacancy formation. This creates localized electronic states that break the extended hybridization of Ce 5d and O 2p orbitals and generates defect levels within the band gap. Electron hopping between Ce^3+^ and Ce^4+^ is enabled by the resultant hybridized Ce 4f–O 2p interaction. In complex oxides, this is expected to enhance the conductivity via electron transport by polaron hopping under reduction conditions. Theoretical investigations also suggest that the interaction between 4f, 5d, and O 2p orbitals is of significance in the description of the defect-mediated properties of cerium oxides.^26^ Mn 2p spectra (Figure 3c,d) show Mn 2p_1/2_ and Mn 2p_3/2_ peaks separated by ∼ 11.8 eV, consistent with manganese oxides.^27^ Deconvolution confirms the presence of mixed valence states of the Mn ions, including their characteristic satellite features (denoted as “sat.”) associated with shakeup transitions during photoemission.^28^ The existence of the mixed valence state in both films is essential to maintain charge neutrality in the presence of oxygen vacancies and electron transport by small-polaron hopping between the Mn^3+^ and Mn^4+^ sites. The Mn^4+^/Mn^3+^ ratio in the two films is quite close (within the error limit) due to the identical deposition conditions for the LB layers in both films. It suggests that the differences in oxygen vacancy concentrations (discussed below) are influenced by the CeO_2_ insertion layer rather than changes in the valence state of manganese. Figure 3e,f presents the O 1s core-level spectra for LB and LBC films. Deconvolution reveals three components at binding energies of ∼ 529.7 eV (O_L_), ∼ 531.0 eV (O_D_), and ∼ 531.9 eV (O_A_), corresponding to lattice oxygen, oxygen deficit regions within the metal oxide matrix, and surface-adsorbed oxygen species, respectively.^29,30^ The peak positions of each elemental feature are summarized in Table 1. The O_L_/O_D_ ratio is lower in the LBC film than the LB film (Table 2), indicating a higher concentration of oxygen vacancies in the LBC films, and thus suggesting that the CeO_2_ layer serves as an oxygen reservoir.

### 3.2 Resistive Switching Characteristics

Current–voltage (I–V) sweeps were performed to characterize the LB and LBC thin-film devices with a compliance current limit of 10 mA. Figures 4a and b show the characteristic I–V loops under a sweeping bias of 0 V → + 1 V → 0 V → – 1 V → 0 V, showing pinched hysteresis loops. As the I–V curves reveal, the electroforming process to activate the resistive switching^31^ requiring a one-time high voltage to form conducting filaments observed as a sudden increase in current, occurs at ∼ 2.8 V for LB and ∼ 2.2 V for LBC (insets). This reduction in forming voltage in LBC can be attributed to the CeO_2_ layer, which enhances oxygen ion migration and reduces the activation energy for conductive filament formation. The I–V curves clearly show transitions between high-resistance states (HRS) and low-resistance states (LRS) as the voltage sweeps from positive to negative and back. Both devices exhibit bipolar resistive switching characteristics with low voltage switching (<1 V). As seen in Figure 4a, the LB device exhibits a small hysteresis window for resistive switching and undefined reset (LRS to HRS transition) voltages, indicating nonuniform filamentary dynamics. In contrast, the LBC device (Figure 4b) shows a larger hysteresis window with a sharp and well-defined set (HRS to LRS transition) and reset voltages. Ten consecutive switching cycles on both LB and LBC devices are shown in Figure S1 of the Supporting Information to evaluate cycle-to-cycle variability. The statistical distribution of the set and reset voltages in the LBC device confirms its stability (Figure S1c in the Supporting Information). Figure 4c shows butterfly hysteresis curves as a function of applied voltage for both devices, confirming nonvolatile bipolar switching. It is worth noting that the ON/OFF (I_LRS_/I_HRS_) ratio at a read voltage of 0.1 V for the LBC device is 2 orders of magnitude higher than that of the LB device under positive or negative biases. For low-power memristor and synapse applications, this value represents a baseline; lower switching ratios (like in the LB device) would have adverse effects on applications. Moreover, this moderate ON/OFF ratio of ∼ 4.6 × 10^2^ of the LBC device is further compensated by the stability of the device. Under positive and negative biases, the different oxygen-ion migration kinetics and interface barrier profiles across the CeO_2_/LBMO junction lead to distinct filament growth and rupture dynamics. This results in differences in the switching magnitudes between the positive and negative polarities. These measurements give evidence that the CeO_2_ layer insertion in the LBC film leads to enhanced growth of conductive filaments through oxygen vacancy stabilization and Ce 4f–O 2p hybridization-dependent electron conduction. The CeO_2_ layer in the junction, acting as a reservoir of oxygen, overcomes the filament destabilization^32^ problem, and leads to a low set voltage and a large ON/OFF ratio in LBC.

**Figure 4:**
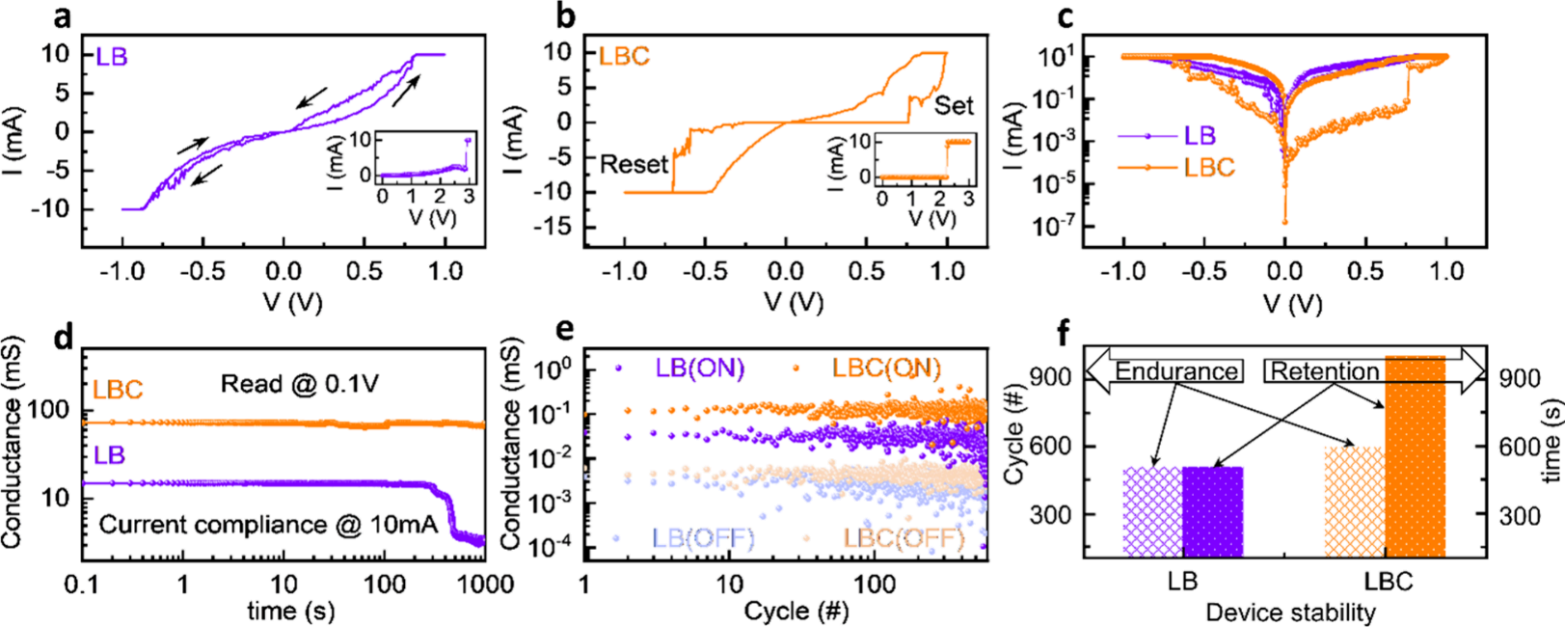
I–V characteristics of (a) LB and (b) LBC, with electroforming curves shown in the insets, (c) butterfly curves illustrating hysteresis behavior, (d) data retention characteristics of the ON state, (e) endurance performance of the devices, (f) device stability.

To assess the stability of the LRS after set transitions, we performed data retention tests for the LB and LBC devices for a sufficiently long-time of 1000 s. Figure 4d shows data retention measurements under constant voltage stress (+0.1 V) as a function of time for LB and LBC. Data retention of the LRS (ON state) was performed for both devices since HRS is the device’s natural state.^33^ The LB device displays partial drift over extended intervals and failed after 500 s, while the LBC device maintains a stable conductance response throughout the measurement range with no observable degradation or failure. The statistical analysis with standard deviation error bars confirms the stability of the LBC device compared to the LB device (See Figure S2 in the Supporting Information). This suggests that the CeO_2_ layer successfully stabilizes oxygen vacancy profiles and maintains filamentary pathways for a longer duration, leading to higher retention of the LBC device. Nonvolatility is verified by stable hysteresis/butterfly I–V loops and endurance (next discussion), complementing the LRS retention results. To evaluate the ability of LB and LBC devices to withstand repeated switching cycles without significant performance degradation, we conducted endurance measurements, shown in Figure 4e. A sequence of triangular pulsed voltages (±1 V, 10 μs width) was applied for 600 switching cycles with a read voltage of 0.3 V. The LB film showed average endurance with gradual degradation of the LRS-to-HRS resistance window after ∼ 500 cycles. In comparison, the LBC film shows stable endurance with a clear LRS and HRS resistance window, indicating that the CeO_2_ layer promotes enhanced performance due to durable and repeatable conductive filaments. The LBC film showed endurance up to 600 cycles without any degradation. Using a ± 2σ deviation criterion, we obtained soft-error rate (SER) values of 2.33% (LB-ON), 2.67% (LB-OFF), 0.33% (LBC-ON), and 1.83% (LBC-OFF), confirming the reduced soft-errors in the LBC device. We used SER as a comparative metric, under which LBC shows improved stability. Thus, the retention and endurance properties, summarized in Figure 4f, demonstrate that the LBC device outperforms the LB device, suggesting that interface-induced stability can lead to creating stable memristive components. It is also worth noting that both LB and LBC show low voltage switching parameters compared with Manganite-based and other bilayer ReRAMs memristors (Table S1 in the Supporting Information). The low-power operation of the present work is pivotal for energy-efficient neuromorphic computing and Internet of Things (IoT) applications. The LBC device is one of the few manganite-based systems validated for neuromorphic applications^34,35^ (discussed in Section 3.3). Overall, the LBC device shows a well-balanced power, performance, and area (PPA) profile, with notable power efficiency and neuromorphic applicability.

In addition to the detailed switching characteristics elaborated above, we show cumulative probability plots of our samples in Figure 5a,b. The plots exhibit data on the cycle-to-cycle variability and reliability of the LB and LBC devices, derived from the endurance data, and reflect the statistical distribution of conductance values for the ON (LRS) and OFF (HRS) states over repeated switching cycles. The LB device has a broad spread of ON and OFF states (Figure 5a), indicating nonuniform filament formation and dissolution during switching cycles. The higher spread in the ON state could be attributed to inconsistencies in achieving uniform conductive filament paths, while partial filament retention contributes to the dispersion of OFF-state values. This is strikingly improved in the case of the LBC device (Figure 5b) that shows narrower distributions for both the ON and OFF states, reflecting more uniform and efficient set-reset processes than the LB device. The reduced cycle-to-cycle variation in the LBC device can be further attributed to the consistent filament dynamics with uniform oxygen vacancy distribution and stabilized charge transport.

**Figure 5:**
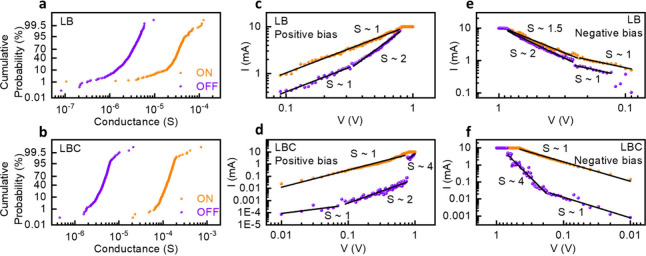
Cumulative probability distributions of (a) LB and (b) LBC; log–log plot fittings for conduction mechanisms under (c) LB positive bias, (d) LBC positive bias, (e) LB negative bias, (f) LBC negative bias.

The log–log I–V data for both HRS and LRS under positive and negative biases were fitted with linear regression over selected voltage regions, as shown in Figure 5c–f. The slopes from these fittings elucidate the charge transport mechanisms in the LB and LBC devices. For the LB device (Figure 5c) under positive bias, the HRS shows a slope (S) of ∼ 1 at low voltage, that can be linked to injected charge carriers being dominated by thermally activated charge transport.^36^ As the voltage increases, the increased slope ∼ 2 indicates the onset of space-charge-limited (SCL) conduction. At this point, the injected charge carriers overcome the thermally generated charge carriers. The LRS shows a consistent slope of ∼ 1, reflecting Ohmic conduction through filamentary paths. On the other hand, in the LBC device (Figure 5d), the HRS slope transitions from ∼ 1 to ∼ 2, then to ∼ 4 at higher voltages, suggesting the presence of trap-filled limit regions and robust space-charge effects. Log–log I–V fittings for the LBC in HRS (positive bias) show low R^2^ values (60.67% for the low-voltage region, 72.44% for the high-voltage region; see Table 3) due to measurement noise and mixed conduction mechanisms in the low-voltage region and complex filament dynamics close to the set transition in the high-voltage region. However, the slopes (∼1 and ∼ 4) confirm thermally activated and trap-filled SCL conduction, respectively, supporting the role of CeO_2_ in stabilizing filamentary conduction. The LRS maintains a slope of ∼ 1. The well-defined SCL regions and higher slope values of the HRS in the LBC device under positive bias highlight its controlled conduction mechanisms, owing to the CeO_2_ layer-induced localized electron transport within the filamentary regions. The LB device has distinct properties under negative bias, as indicated in Figure 5e. The LRS has a low-voltage slope of ∼ 1 and a high-voltage slope of ∼ 1.5. This results from the absence of a stabilizing oxide such as CeO_2_, leading to asymmetric filament dynamics. The HRS has a slope of ∼ 2 at high voltages, indicative of filament rupture during reset operation. In the low-voltage region, the HRS slope is still ∼ 1, as expected in thermally activated residual carriers-dominated conduction following filament reset. The LRS with a single-fitting region with a slope of ∼ 1 for the LBC device in negative bias (Figure 5f) implies stable Ohmic conduction through robust and uniform filaments. In HRS, the slope of ∼ 4 reflects more control over filamentary processes. The similar LRS slopes and steeper HRS slopes in LBC signify the ability of the CeO_2_ layer to promote controlled diffusion, enabling homogeneous and reversible filament growth.

**Table 3:** R^2^ Values of LB and LBC Devices from Log–Log Fittings of Figure 5 ^a^

Sample		Positive bias				Negative bias			
LB	Slope	S1 (∼1)	S2 (∼2)	S3 (∼1)		S4 (∼1)	S5 (∼1.5)	S6 (∼2)	S7 (∼1)
	R^2^	96.29	98.96	99.17		94.75	97.94	98.68	93.56
LBC	Slope	S1 (∼1)	S2 (∼2)	S3 (∼4)	S4 (∼1)	S5 (∼1)	S6 (∼4)	S7 (∼1)	
	R^2^	60.67	92.68	72.44	97.46	99.39	93.18	91.81	

a Slope numbers (slope 1, slope 2, slope 3, and so on) correspond to segments of the voltage sweep sequence: 0 V → + 1 V → 0 V → −1 V → 0 V.

Furthermore, I–V curves were performed on three LBC devices, as shown in Figure 6a, to highlight the device-to-device variability. Forming, set, and reset voltages of the three devices, along with corresponding error bars, are shown in Figure 6b. The consistent forming and switching voltages observed across the devices demonstrate good reproducibility. Endurance plots of three LBC devices confirm the device-to-device stability (see Figure S3 in the Supporting Information). To investigate the thermal stability of the LBC device (d1 LBC device was selected for all studies), we performed switching measurements as shown in Figure 6c. The switching window shows a very minute reduction at 80 °C. Log–log I–V fittings at different temperatures (Figure S4; Table 4) show an Ohmic conduction in LRS with a slope of ∼ 1 in both polarities. At 80 °C, the HRS shows characteristic multiregion SCLC behavior with slopes of ∼ 1 to ∼ 2 and ∼ 3 at positive biases. The R^2^ values are slightly reduced at high voltages due to thermal noise, however the extracted slope values are consistent with trap-filled SCLC conduction. In negative bias, the LRS remains Ohmic, and the HRS shows a steep slope of ∼ 4, suggesting controlled filament rupture. These results confirm the validity of log–log fittings under thermal conditions. Furthermore, it confirms that the CeO_2_ layer maintains stable filamentary transport with SCLC behavior up to a studied temperature of 80 °C, which is suitable for most practical applications. The structural, spectroscopic, and electrical measurement results reliably complement each other, indicating the CeO_2_-controlled, oxygen-vacancy-mediated filament formation and stabilization in the LBC device.

**Figure 6:**
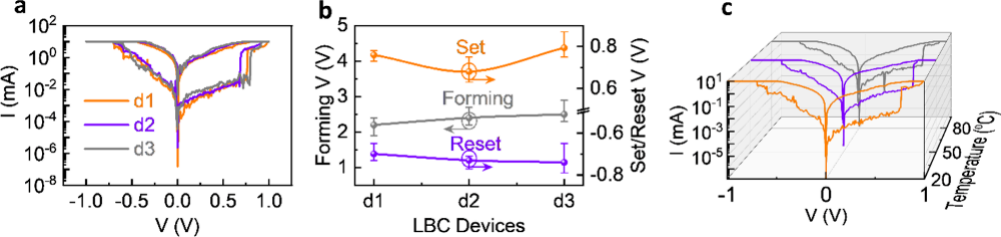
(a) Butterfly curves of LBC devices, (b) forming, set, and reset voltages of LBC devices with corresponding error bars, (c) temperature-dependent hysteresis characteristics of LBC device d1.

**Table 4:** Temperature-Dependent Fitting Slope Values of the LBC Device (from Figure S4)

Temperature (°C)		Positive bias					Negative bias		
20	Slope	S1 (∼1)	S2 (∼2)	S3 (∼4)	S4 (∼1)		S5 (∼1)	S6 (∼4)	S7 (∼1)
	R^2^	60.67	92.68	72.44	97.46		99.39	93.18	91.81
50	Slope	S1 (∼1)	S2 (∼2)	S3 (∼6)	S4 (∼1)		S5 (∼1)	S6 (∼4)	S7 (∼1)
	R^2^	96.46	97.75	99.81	96.63		99.26	97.76	96.28
80	Slope	S1 (∼1)	S2 (∼2)	S3 (∼3)	S4 (∼2)	S5 (∼1)	S6 (∼1)	S7 (∼4)	S8 (∼1)
	R^2^	95.67	80.81	66.36	99.44	98.28	99.54	97.20	91.01

### 3.3 Resistive Switching Mechanism and Synapse Characteristics

Considering the XPS analysis, here we present a mechanism of conduction in the films, governed by the interaction of oxygen vacancies with electron orbitals and charge carriers. The resistive switching dynamics are schematically depicted in Figure 7a,b. In the LB device (Figure 7a), under a positive bias to the top electrode during the set process (left panel), oxygen ions are pushed out of the LBMO layer and toward the top electrode. The migration of oxygen ions leaves behind oxygen vacancies in the LBMO layer. Modification of local electronic structures close to the Fermi level is achieved by these oxygen vacancies acting as localized electron traps. With increased oxygen vacancy density, their overlapping electronic states promote the formation of a discontinuous, thin metallic conductive filament. The device switches to an ON state by these filaments connecting the top and bottom electrodes. The redox activity of Mn^3+^/Mn^4+^ supports these filaments and provides a low-resistance path with predominantly Ohmic conduction. Electron transport occurs via polaron hopping between Mn 3d and O 2p orbitals, whose interaction is enhanced by the local structural distortion caused by oxygen vacancies. In the reset stage, when the top electrode is given a negative bias (right side of Figure 7a), the electric field drives the oxygen ions back into the LBMO. This partial reintegration of oxygen ions into the lattice breaks the conductive filament and restores the device to an OFF state. However, the lack of a stabilizing oxygen reservoir layer, i.e., CeO_2_, results in nonuniform reintegration of oxygen ions. Thus, filament breakdown is nonuniform and causes more randomness and less stable switching. The XPS measurements confirm that electronic states of oxygen vacancies in the LB device have no stabilizing hybridization with higher energy orbitals. Therefore, the phenomenon of switching has a nonuniform nature.

**Figure 7:**
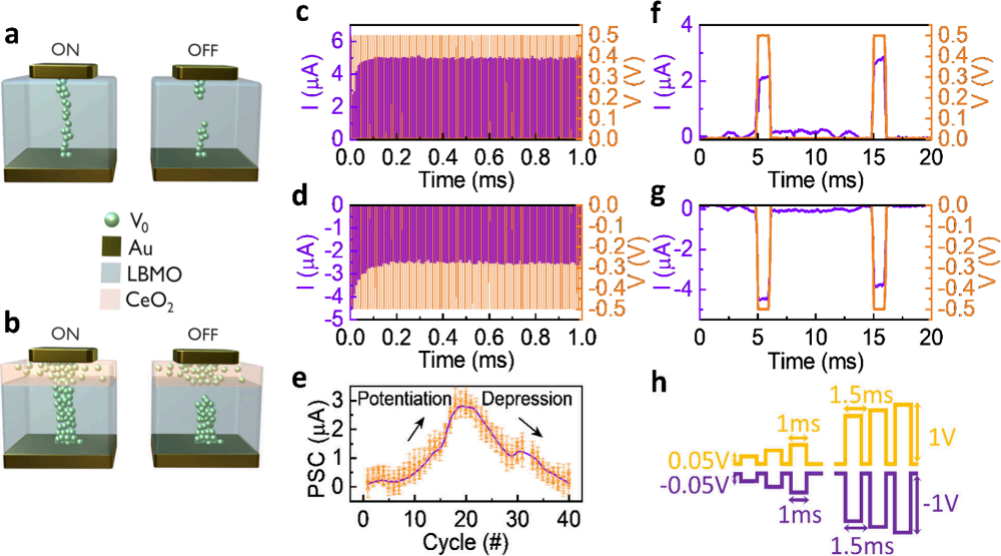
Schematic representation of switching mechanism in (a) LB and (b) LBC; dynamic response of LBC film showing (c) synapse potentiation and (d) synapse depression; (e) potentiation and depression using nonidentical pulses, repeated six times, with corresponding standard deviation; implementation of one pair of cycles on LBC device in (f) positive bias and (g) negative bias; (h) potentiation and depression pulse scheme for LBC device consisting of 40 pulses, every pulse followed with 100 mV read voltage.

On the other hand, the LBC device (Figure 7b) displays improved resistive switching kinetics because of the incorporation of the CeO_2_ oxygen reservoir layer. Applying an external positive bias in the set process (left panel) enables the movement of oxygen ions from the LBMO switching layer to the CeO_2_ layer. The CeO_2_ layer acts as an effective reservoir for mobile O^2–^ ions in the presence of a high concentration of oxygen vacancies (confirmed by XPS analysis). This oxygen ion migration creates a dense network of oxygen vacancies in the LBMO layer, promoting the formation of a continuous and robust conductive filament, switching the device to the ON state. The filamentary conduction is enabled by polaron hopping between Mn^3+^ and Mn^4+^ ions, supported by localized states within the bandgap. The redox activity of Ce^4+^/Ce^3+^ in the CeO_2_ layer lowers the Fermi level of LBMO closer to the conduction band minimum and promotes strong hybridization between Ce 4f and O 2p orbitals. This orbital coupling stabilizes the oxygen ions, mitigating the overdiffusion of vacancies and thus providing stability and homogeneity to filaments. During the reset process (right panel of Figure 7b), a negative bias drives oxygen ions back from the CeO_2_ layer into the LBMO switching layer. The controlled migration of the oxygen ions breaks the conductive filament by occupying oxygen vacancy sites, restoring a wider bandgap-like electronic structure. The energy barrier at the CeO_2_/LBMO interface is restored, preventing the carrier transport, leading to switching the LBC device back to the OFF state. This controlled switching dynamic stabilization prevents filament damage and makes resistive switching reversible and reliable across cycles in LBC.

Having established the stable LBC performance with a larger switching window, lower operating voltage, improved retention, and enhanced endurance, we now turn our attention to synaptic performance. Learning (potentiation) and forgetting (depression) properties are depicted in Figures 7c and 7d for the LBC device. A pulse train with a 1 ms pulse width, 10 ms time interval, and 0.5 V amplitude was applied to the LBC device, and the device conductance was recorded after each pulse to quantify the incremental changes in conductance, representing synaptic weight modulation. A characteristic pair of such pulses for potentiation and depression is shown in Figure 7f and g. Figure 7c shows a gradual increase in device conductance with successive pulse stimuli under a positive bias, indicating potentiation. Subsequent positive pulses drive oxygen ions from the LBMO layer to the CeO_2_ reservoir, enhancing vacancy accumulation, strengthening the filament, and increasing conductance incrementally and stepwise synaptic weight modulation reflecting biological synaptic potentiation. Figure 7d illustrates depression, with a stepwise reduction in conductance with multiple pulse stimuli under negative bias. The negative bias pulse train with identical pulse parameters was applied right after the positive pulse train, as reported in a prior study.^37^ Successive negative pulses drive oxygen ions back into the LBMO layer, partially refilling vacancies and thinning the filament stepwise, leading to a progressive decrease in conductance. This process demonstrates synaptic weakening, which is essential for implementing forgetting in neuromorphic systems. The programming energy per pulse was ≈ 200 pJ per event, consistent with recently reported CeO_2_-based memristors^38^ and within the typical range for oxide-based analog devices, confirming the energy-efficient nature of the LBC device.

Synaptic behaviors for potentiation and depression using nonidentical pulse trains are shown in Figure 7e. We used nonidentical pulse trains since they enhance analog linearity, consistent with previous report.^39^ A sequence of 20 positive pulses followed by 20 negative pulses, with linearly varying amplitudes (0.05 to 1 V), 1 ms width, and 1.5 ms interval, was applied to the LBC device (pulse scheme in Figure 7h, consistent with literature^40^). In potentiation, postsynaptic current (PSC) rises while amplitudes of positive pulses increase, showing the development of synaptic contacts similar to biological organisms. During potentiation, the CeO_2_ layer traps oxygen ions. The growing amplitude in a linear trend shows the decrease in PSC in the depression. This phenomenon is representative of synaptic weakening, as seen in biological systems. In the depression, oxygen ions are sourced by the CeO_2_ layer. To quantify repeatability between consecutive cycles, the cycle-to-cycle correlation coefficient, defined as the Pearson correlation coefficient (r),^41^ was used. The coefficient r for potentiation (average) is 0.98, and for depression (average) is 0.97. Across all six potentiation and six depression repetition, the correlation between successive cycles remains consistently above 0.96, demonstrating that the conductance evolution is highly stable and exhibits minimal stochastic drift. For ideal synaptic devices, a linear change of potentiation and depression is desired. The asymmetric ratio (AR), the conductance change between potentiation and depression, is given by,^42^

2 $$AR=\frac{max\left|G_{p}\left(n\right)-G_{d}\left(n\right)\right|}{G_{p}\left(n_{\max}\right)-G_{d}\left(n_{\max}\right)}$$

where, *G*_*p*_(*n*) is channel conductance after the *n* ^th^ potentiation pulse, *G*_*d*_(*n*) is channel conductance after the *n* ^th^ depression pulse, *G*_*p*_(*n*_*max*_) is channel conductance after the last potentiation pulse, *G*_*d*_(*n*_*max*_) is channel conductance after the last depression pulse, *n*_*max*_ is the total number of pulses in one phase (potentiation or depression). The AR should be zero for an ideal symmetry.^42^ The AR of the LBC device is estimated to be 0.298 ± 0.093 (refer to Table S2 in the Supporting Information for the normalized PSC pertaining to AR calculation), which shows reasonable symmetry close to the ideal case. Our device demonstrates nonlinear but symmetric behavior, which aligns with Gokmen et al.,^43^ who show that such behaviors support neural network training by adjusting the learning rate without harming the optimization process. The DC I–V characteristics show smooth hysteresis and gradual, stable, and controllable conductance changes, while the synaptic pulse responses under AC stimulation directly correlate with these DC behaviors. Together, DC and AC behaviors confirm gradual conductance modulation, suggesting intrinsic analog tunability and multibit storage potential. The gradual potentiation and depression effects in the LBC device represent biological synaptic plasticity and reliable low-voltage performance, representing it as a suitable candidate for neuromorphic and practical applications beyond fundamental characterizations.

## 4 Conclusion

In this work, we demonstrated the impact of interface engineering in LBMO devices with high-performance resistive switching. Through detailed transport and electronic characterization, we established that the LBC device involving CeO_2_ oxygen reservoir achieves low voltage resistive switching and 2 orders of magnitude enhancement in the ON/OFF ratio with superior endurance and retention. This work also reveals a new insight into the conduction mechanism that the CeO_2_ layer provides high oxygen vacancy defects by the redox process between Ce^4+^ and Ce^3+^ observed in XPS, providing stable conducting filament paths in the LBC device. The LBC device shows biologically similar potentiation and depression responses close to ideal symmetry. The CeO_2_/LBMO structure shows a good mix of endurance and energy efficiency, making it suitable for adaptive and intelligent hardware platforms. Our results reveal that this reservoir junction–based memristor achieves stable conduction and dependable analog switching. It lays a materials foundation for scalable, low-power in-memory and neuromorphic computing designs controlled by oxygen-vacancy dynamics.

## Supplementary Material



## Notes

Ten switching cycles for the LB and LBC devices, LBC set/reset voltage distribution in the LBC device, retention time error, endurance of LBC devices, temperature-dependent SCL fittings in the LBC device, manganite-based memristors comparison, and PSC for asymmetric ratio (PDF)
