# Supplementary material for: Interface-Induced Synaptic Performance in CeO2/La0.8Ba0.2MnO3 Oxygen Reservoir Junction
Source: ACS Appl Mater Interfaces. 2025 Dec 10;17(51):69666–75. doi: 10.1021/acsami.5c19731 (PMC12754756; doi:10.1021/acsami.5c19731)
Supplement: Supplementary file 1 [file am5c19731_si_001.pdf]

Supporting information

Interface-Induced Synaptic Performance in $CeO_2/La_{0.8}Ba_{0.2}MnO_3$ Oxygen Reservoir Junction

K. N. Rathod[1]*, Gopal Datt[2], Bagher Aslibeiki[3,1], Ted Johansson[4], Gianni Barucca[5], Davide Peddis[6,7], M. Venkata Kamalakar[2]*, Tapati Sarkar[1]*

[1]Division of Solid-State Physics, Department of Materials Science and Engineering, Uppsala University, Uppsala SE-751 03, Sweden

[2]Division of X-ray Photon Science, Department of Physics and Astronomy, Uppsala University, Uppsala SE-751 20, Sweden

[3]Faculty of Physics, University of Tabriz, Tabriz 51666-16471, East Azerbaijan Province, Tabriz, Iran

[4]Division of Solid-State Electronics, Department of Electrical Engineering, Uppsala University, Uppsala SE-751 03, Sweden

[5]Department of Science and Engineering of Matter, Environment and Urban Planning, University Politecnica delle Marche, Via Brecce Bianche 12, 60131 Ancona, Italy

[6]Department of Chemistry and Industrial Chemistry & Genova INSTM RU, nM2-Lab, University of Genova, 16146 Genova, Italy

[7]National Research Council, Institute of Structure of Matter, nM2-Lab, Via Salaria km 29.300, Monterotondo Scalo 00015, Roma, Italy

*kunalsinh.rathod@angstrom.uu.se, *venkata.mutta@physics.uu.se,
*tapati.sarkar@angstrom.uu.se

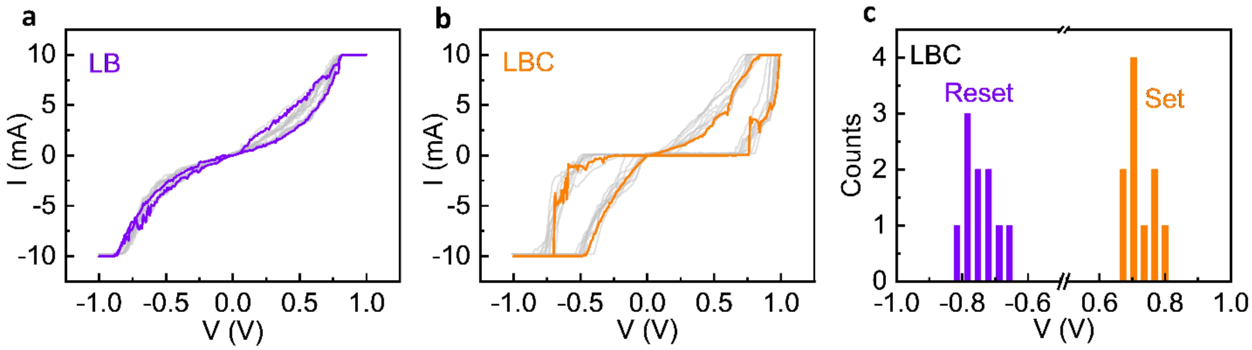

Figure S1. Ten subsequent switching cycles with the same current compliance of 10 mA in (a) LB and (b) LBC, (c) Set/reset voltage statistical distribution of LBC device.

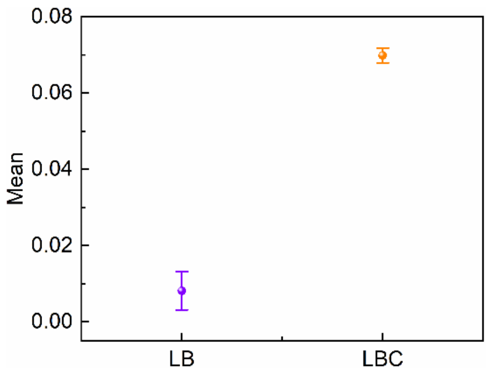

Figure S2. Statistical analysis of retention time with error bars for LB and LBC devices, corresponding to the data presented in Figure 4d.

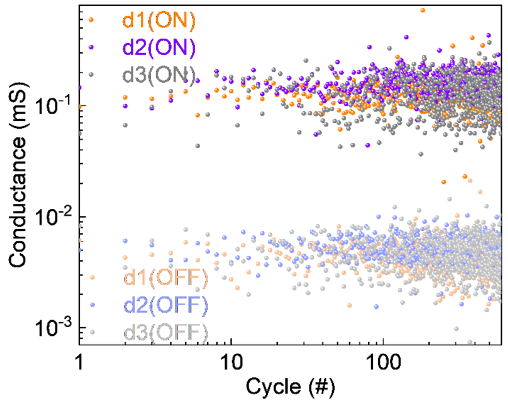

Figure S3. Endurance plot for three LBC devices.

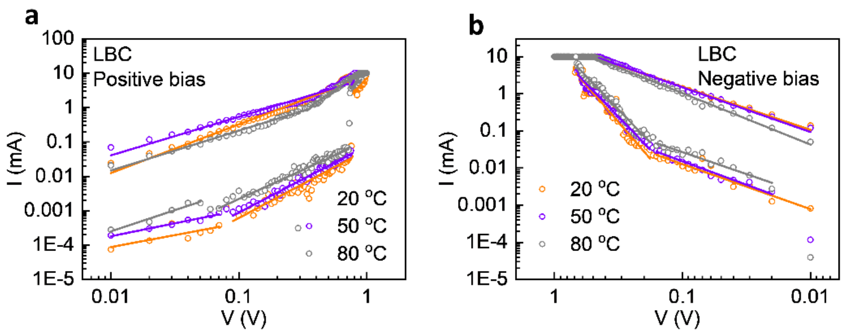

Figure S4. Temperature dependence of SCL fittings in the LBC device d1 of Figure 6c under (a) positive bias and (b) negative bias.

Table S1. Comparison of manganite-based and other bilayer memristors.

Sample	Power		Performance			Cell/electrode area (μm^2)	ML/ MS	N_{App}	Ref.
	V_F	V_S	R_S	R_t	E				
$La_{2/3}Ba_{1/3}MnO_3$	-	~2.6	-	~10^4	-	2×2	Yes	No	1
$Pr_{0.7}Ca_{0.3}MnO_3$/GO	FF	0.75	10^2	10^4	1.5×10^2	-	NR	No	2
$Pr_{0.7}Ca_{0.3}MnO_3$	~4	-	10^3	-	-	-	NR	No	3
$La_{2/3}Ba_{1/3}MnO_3$	-	-	-	10^5	3×10^3	3×3	Yes	No	4
$La_{2/3}Ba_{1/3}MnO_3$	FF	~2.7	30	10^4	10^4	3×3	NR	No	5
$Pr_{0.7}Ca_{0.3}MnO_3/AlO_x$	FF	1.5	10^2	10^4	100	250nm (diameter)	NR	No	6
TaN/HfO_2/Al_2O_3/ITO	~4.5	~1.5	~10^2	>2×10^3	>100	-	Yes	Yes	7
FeNi/Al_2O_3/NiO/Pt	~4.07	~6.0	~10^3	>10^4	>100	-	NR	No	8
Au/TiO_x/TiO_y/Au	~5.62	~1.0	~10^2	-	-	20 × 20	Yes	Yes	9
Pt/Hf/HfO_2/TiN	FF	~0.8	~10^2	>10^6	-	-	NR	No	10
Al_2O_3/ Ru NCs	-	1	>10^5	10^5	-	-	NR	No	11
TiO_x/MgO	FF	1.4	< 32	-	>10^3	50 × 50	Yes	No	12
WO_3/Al_2O_3	3	3.5	10^4	-	300	-	NR	No	13
$La_{0.8}Sr_{0.2}MnO_3$/ CeO_2	~4.2	1	~10^3	-	10^2	-	NR	No	14
$La_{0.7}(Sr_{0.1}Ca_{0.9})_{0.3}MnO_3/CeO_2$	-	1.8	~10^3	-	-	<1×10^6	NR	No	15
$La_{0.8}Ba_{0.2}MnO_3$	2.9	0.8	2.2	500	500	~1×10^6	No	No	**This work**
$La_{0.8}Ba_{0.2}MnO_3/CeO_2$	2.2	0.76	459	10^3	600	~1×10^6	Yes	Yes	**This work**

V_F = Forming voltage (V), V_S = Set voltage (V), R_S = ON/OFF ratio, R_t = Retention (s), E = Endurance (#cycles), ML = multi-level, MS = multi-state, N_{App} = Neuromorphic application, FF = Forming free, NR = not reported. A comparative benchmark table summarizing performance, power, and area (PPA) metrics of the work against other bilayer ReRAM studies.

PSC was normalized for pulses 1 to 20 (potentiation) and 21 to 40 (depression). Mapping pulses 40 to 21 for depression with 1 to 20 potentiation pulses to calculate the asymmetry ratio (AR), since the depression should have similar conductance as the potentiation.

Table S2. PSC of potentiation and depression after normalization for the calculation of the asymmetric ratio.

n	$\boldsymbol{G_p(n)}$ (pulse)	$\boldsymbol{G_d(n)}$ (pulse)	$\boldsymbol{\lvert G_p(n) - G_d(n) \rvert}$
1	0 (1)	0 (pulse 40)	0
2	0.0259 (2)	0.04638 (pulse 39)	0.02048
3	0.04179 (3)	0.07851 (pulse 38)	0.03672
4	0.04765 (4)	0.12874 (pulse 37)	0.08109
5	0.0248 (5)	0.15318 (pulse 36)	0.12838
6	0.02807 (6)	0.1939 (pulse 35)	0.16583
7	0.02514 (7)	0.29218 (pulse 34)	0.26704
8	0.06569 (8)	0.34959 (pulse 33)	0.2839
9	0.08003 (9)	0.34959 (pulse 32)	0.26956
10	0.13887 (10)	0.41325 (pulse 31)	0.27438
11	0.18839 (11)	0.42659 (pulse 30)	0.2382
12	0.25328 (12)	0.33215 (pulse 29)	0.07887
13	0.34197 (13)	0.3913 (pulse 28)	0.04933
14	0.4226 (14)	0.45755 (pulse 27)	0.03495
15	0.42037 (15)	0.549 (pulse 26)	0.12863
16	0.5886 (16)	0.64587 (pulse 25)	0.05727
17	0.84849 (17)	0.75043 (pulse 24)	0.09806
18	0.92956 (18)	0.82191 (pulse 23)	0.10765
19	1 (19)	0.93929 (pulse 22)	0.06071
20	0.95232 (20)	1 (pulse 21)	0.04768

REFERENCES

(1) Wen, J.; Zhao, X.; Li, Q.; Zhang, S.; Wang, D.; Du, Y. Multilevel Resistance Switching Effect in Au/$La_{2/3}Ba_{1/3}MnO_3$/Pt Heterostructure Manipulated by External Fields. *J Magn Magn Mater* **2018**, *452*, 184–187. https://doi.org/10.1016/j.jmmm.2017.12.081.

(2) Kim, I.; Siddik, M.; Shin, J.; Biju, K. P.; Jung, S.; Hwang, H. Low Temperature Solution-Processed Graphene Oxide/$Pr_{0.7}Ca_{0.3}MnO_3$ Based Resistive-Memory Device. *Appl Phys Lett* **2011**, *99* (4), 042101: 1-3. https://doi.org/10.1063/1.3617426.

(3) Tsubouchi, K.; Ohkubo, I.; Kumigashira, H.; Oshima, M.; Matsumoto, Y.; Itaka, K.; Ohnishi, T.; Lippmaa, M.; Koinuma, H. High-Throughput Characterization of Metal Electrode Performance for Electric-Field-Induced Resistance Switching in Metal/$Pr_{0.7}Ca_{0.3}MnO_3$/Metal Structures. *Advanced Materials* **2007**, *19* (13), 1711–1713. https://doi.org/10.1002/adma.200601957.

(4) Zhou, W.; Xiong, Y.; Zhang, Z.; Wang, D.; Tan, W.; Cao, Q.; Qian, Z.; Du, Y. Multilevel Resistance Switching Memory in $La_{2/3}Ba_{1/3}MnO_3$/0.7Pb($Mg_{1/3}Nb_{2/3}$)O_3-0.3PbTiO_3 (011) Heterostructure by Combined Straintronics-Spintronics. *ACS Appl Mater Interfaces* **2016**, *8* (8), 5424–5431. https://doi.org/10.1021/acsami.5b11392.

(5) Xiong, Y. Q.; Zhou, W. P.; Li, Q.; Cao, Q. Q.; Tang, T.; Wang, D. H.; Du, Y. W. Electric Field Modification of Magnetism in Au/$La_{2/3}Ba_{1/3}MnO_3$/Pt Device. *Sci Rep* **2015**, *5*, 12766: 1-7. https://doi.org/10.1038/srep12766.

(6) Park, S.; Jung, S.; Siddik, M.; Jo, M.; Lee, J.; Park, J.; Lee, W.; Kim, S.; Sadaf, S. M.; Liu, X.; Hwang, H. Memristive Switching Behavior in $Pr_{0.7}$ $Ca_{0.3}$ MnO_3 by Incorporating an Oxygen-Deficient Layer. *Physica Status Solidi - Rapid Research Letters* **2011**, *5* (10–11), 409–411. https://doi.org/10.1002/pssr.201105317.

(7) Mahata, C.; Lee, C.; An, Y.; Kim, M. H.; Bang, S.; Kim, C. S.; Ryu, J. H.; Kim, S.; Kim, H.; Park, B. G. Resistive Switching and Synaptic Behaviors of an HfO_2/Al_2O_3 Stack on ITO for Neuromorphic Systems. *J Alloys Compd* **2020**, *826*, 154434. https://doi.org/10.1016/j.jallcom.2020.154434.

(8) Wang, G.; Hu, L.; Xia, Y.; Li, Q.; Xu, Q. Resistive Switching in FeNi/Al_2O_3/NiO/Pt Structure with Various Al_2O_3 Layer Thicknesses. *J Magn Magn Mater* **2020**, *493*, 165728:1-7. https://doi.org/10.1016/j.jmmm.2019.165728.

(9) Kim, J.; Cho, S.; Kim, T.; Pak, J. J. Mimicking Synaptic Behaviors with Cross-Point Structured TiO_x/TiO_y-Based Filamentary RRAM for Neuromorphic Applications. *Journal of Electrical Engineering and Technology* **2019**, *14* (2), 869–875. https://doi.org/10.1007/s42835-019-00107-y.

(10) Cai, L.; Chen, W.; Zhao, Y.; Liu, X.; Kang, J.; Zhang, X.; Huang, P. Insight into Effects of Oxygen Reservoir Layer and Operation Schemes on Data Retention of HfO_2-Based RRAM.

IEEE Trans Electron Devices **2019**, *66* (9), 3822–3827. https://doi.org/10.1109/TED.2019.2928626.

(11) Chen, L.; Gou, H. Y.; Sun, Q. Q.; Zhou, P.; Lu, H. L.; Wang, P. F.; Ding, S. J.; Zhang, D. Enhancement of Resistive Switching Characteristics in Al_2O_3-Based RRAM with Embedded Ruthenium Nanocrystals. *IEEE Electron Device Letters* **2011**, *32* (6), 794–796. https://doi.org/10.1109/LED.2011.2125774.

(12) Lu, W.; Chen, W.; Li, Y.; Jha, R. Self Current Limiting MgO ReRAM Devices for Low-Power Non-Volatile Memory Applications. *IEEE J Emerg Sel Top Circuits Syst* **2016**, *6* (2), 163–170. https://doi.org/10.1109/JETCAS.2016.2547758.

(13) Woo, J.; Belmonte, A.; Redolfi, A.; Hwang, H.; Jurczak, M.; Goux, L. Introduction of WO_3 Layer in a Cu-Based Al_2O_3 Conductive Bridge RAM System for Robust Cycling and Large Memory Window. *IEEE Journal of the Electron Devices Society* **2016**, *4* (3), 163–166. https://doi.org/10.1109/JEDS.2016.2526632.

(14) Ortega-Hernandez, R.; Coll, M.; Gonzalez-Rosillo, J.; Palau, A.; Obradors, X.; Miranda, E.; Puig, T.; Suñe, J. Resistive Switching in $CeO_2/La_{0.8}Sr_{0.2}MnO_3$ Bilayer for Non-Volatile Memory Applications. *Microelectron Eng* **2015**, *147*, 37–40. https://doi.org/10.1016/j.mee.2015.04.042.

(15) Chen, X. G.; Fu, J. B.; Liu, S. Q.; Yang, Y. B.; Wang, C. S.; Du, H. L.; Xiong, G. C.; Lian, G. J.; Yang, J. B. Trap-Assisted Tunneling Resistance Switching Effect in $CeO_2/La_{0.7}(Sr_{0.1}Ca_{0.9})_{0.3}MnO_3$ Heterostructure. *Appl Phys Lett* **2012**, *101* (15), 153509: 1-4. https://doi.org/10.1063/1.4760221.
